# Dysbiosis and Therapeutic Modulation of the Gut Microbiota in Multiple Sclerosis: A Narrative Review

**DOI:** 10.1002/hsr2.71564

**Published:** 2025-12-01

**Authors:** Mojtaba Memariani, Hamed Memariani

**Affiliations:** ^1^ Department of Mycobacteriology and Pulmonary Research Pasteur Institute of Iran Tehran Iran; ^2^ Microbiology Research Center (MRC) Pasteur Institute of Iran Tehran Iran; ^3^ Biotechnology Research Center Pasteur Institute of Iran Tehran Iran

**Keywords:** diet, dysbiosis, gut microbiota, multiple sclerosis, probiotics

## Abstract

**Background and Aims:**

Multiple sclerosis (MS) is a persistent autoimmune disease that affects the central nervous system. The etiology of MS is complex, involving a variety of genetic and environmental factors. Mounting evidence suggests that dysbiosis significantly impacts the progression of MS mainly through its direct effects upon the immune system. Given the vital connection between the gut microbiota and immune health, particularly in the context of autoimmune diseases, this review aims to summarize the existing knowledge regarding alterations in the gut microbiota among MS patients, with a focus on microbiota‐based therapeutic approaches.

**Methods:**

A detailed literature review was carried out to gather contemporary evidence on dysbiosis of the gut microbiota in MS patients. Furthermore, studies dealing with the modification of gut microbiota for therapeutic applications in MS have been included.

**Results:**

A distinct variation in specific bacterial phyla, orders, families, and genera, as well as metabolites, was found in MS patients. Exploring therapeutic options such as antibiotics, probiotics, dietary interventions, fecal microbiota transplantation, phage therapy, and helminth therapy may present valuable opportunities for gut microbiota modification in MS treatment.

**Conclusion:**

Altering the gut microbiota in patients with MS may serve as a potentially effective treatment strategy. Nevertheless, future research should prioritize the standardization of these therapies. Finally, it is imperative that researchers concentrate on large‐scale studies or trials to scrutinize the practical relevance of these therapeutic options.

## 1 Introduction

Multiple sclerosis (MS) is a chronic neuroinflammatory and neurodegenerative disease of the central nervous system (CNS). The hallmark of MS is the appearance of disseminated lesions in the brain and spinal cord, characterized by immune‐mediated demyelination and axonal loss [1]. These lesions contribute to the development of various neurological deficits and culminate in the gradual accumulation of disability. The disease can present with a constellation of symptoms such as dizziness, vision loss, vertigo, motor dysfunction, pain, numbness, impaired coordination, fatigue, and depression [2]. The global statistics regarding MS are truly alarming, with roughly 2.8 million individuals grappling with its debilitating ramifications, especially among the youth. A wealth of epidemiological studies has also adduced plentiful evidence in favor of the female preponderance of MS [3]. There exist various forms of MS, each differentiated by unique disease progression and disability symptoms. These forms involve relapsing–remitting MS (RRMS), secondary progressive MS (SPMS), primary progressive MS (PPMS), and progressive relapsing MS (PRMS). The most prevalent form is RRMS, in which there are intermittent periods of remission and inflammatory relapses [4].

The etiology of MS continues to be a subject of ongoing research scrutiny, with no decisive conclusions drawn yet. Nevertheless, it is a reasonable conjecture that a complex interplay of factors (e.g., gender, genetic variables, and environmental factors) may play a pivotal part in its onset. Smoking, vitamin D level, intestinal microbiota status, and Epstein–Barr virus (EBV) are thought to be the major culprits behind the MS development [5]. However, risk factors such as lifestyle choices and obesity are still considered speculative and deserve further illumination to establish their plausible role in the disease [6].

MS is characterized by the infiltration of immune cells from the periphery into the CNS. The pathogenesis of MS is marked by the existence of pro‐inflammatory self‐reactive T cells, including T‐helper 1 (Th1), Th17, and Th22 cells producing interferon gamma (IFN‐γ), interleukin 17 (IL‐17), and IL‐22. Furthermore, a deficiency in regulatory T cells (Tregs) is observed, resulting in their compromised ability to suppress the autoreactive immune response. The contribution of B lymphocytes to the inflammatory mechanisms in the CNS is notable, as they generate autoantibodies, present antigens, and modulate T cell responses. An imbalance between autoreactive and regulatory elements can culminate in the development of demyelinating lesions in the CNS [7]. Among the chief symptoms manifested by those suffering from MS are motor symptoms, including tremors and paroxysmal dyskinesias, as well as visual symptoms like optic neuritis. Furthermore, cognitive and mental deterioration often accompany disease exacerbations [8].

A paradigm shift has occurred in the field of biomedicine over the past decades. It has become increasingly clear that the gut microbiota is indispensable for upholding homeostasis and regulating nearly all major body systems. Burgeoning evidence accentuates the role of the gut microbiota in key aspects of neurological development, neuroinflammation, and behavior [9]. The gut‐brain axis concept is now gaining traction as it seeks to demystify the mutual influence between two anatomically distinct biological systems. The imbalance of this axis has been implicated in the pathogenesis of a broad swath of neurological disorders, including, but not restricted to, Alzheimer′s disease (AD), Parkinson′s disease (PD), Huntington′s disease (HD), autism spectrum disorder (ASD), and MS [10]. The intention of this review is to summarize the current knowledge germane to the gut microbiota alterations in MS while also highlighting the recent advancements in microbiota‐oriented therapeutic strategies.

## 2 Methods

A comprehensive literature review was performed to collect current evidence regarding gut microbiota dysbiosis in MS patients, as well as studies that focused on altering gut microbiota through different therapeutic approaches for MS treatment. The literature for this review was obtained through an electronic search of databases including PubMed/MEDLINE, EMBASE, Google Scholar, and Scopus, using search terms including “multiple sclerosis” AND “gut dysbiosis” OR “antibiotics” OR “probiotics” OR “prebiotics” OR “synbiotics” OR “postbiotics” OR “psychobiotics” OR “antimicrobial agents” OR “diet” OR “fecal microbiota transplantation” OR “phage therapy” OR “helminth therapy”. The present review incorporated studies published from 2000 onward, with a focus on more recent articles to capture the latest research and advancements. Only articles written in English were selected for inclusion. Publications lacking detailed methodologies or containing redundant information were excluded from the review.

## 3 Pathophysiology of MS

As mentioned earlier, the underlying cause of MS is still uncertain, but data suggest that its pathophysiology includes two significant and interrelated factors: neuroinflammation and neurodegeneration. The inflammatory aspect of MS pathophysiology involves an aberrant activation of the immune system against CNS antigens, resulting in the interaction of autoreactive leukocytes with the inflamed cerebral endothelium, the disruption of the blood‐brain barrier (BBB), and the migration of these activated leukocytes into the CNS parenchyma [11]. The early catalysts that incite these vigorous immune responses and leukocyte activation against self‐antigens are not easily discernible; however, viral and bacterial antigens are likely significant triggers of the underlying MS pathophysiology. It has been proposed that exposure to or infection by several viruses, such as hepatitis B and EBV, activates T lymphocytes that recognize viral proteins with structural similarities to CNS proteins, including myelin basic protein (MBP); this concept is known as the “molecular mimicry hypothesis” [12].

The pathophysiology of MS encompasses both the innate and adaptive immune systems. A proposed explanation for the pathophysiology of MS is that the process commences in the peripheral circulation, where immune cells, including dendritic cells (DCs), are activated outside the CNS. Animal studies have uncovered that autoreactive T lymphocytes (CD4^+^ and CD8^+^) specific to CNS antigens are key players in the development of demyelinating lesions in the CNS. Through firm binding and rolling, autoreactive T lymphocytes and monocytes engage with inflammatory cerebral endothelial cells in the cerebrovascular space. The leukocytes are committed to migrating across postcapillary venules into the CNS. This binding process is the most important part of the leukocyte–endothelial interaction. When autoreactive T cells and monocytes infiltrate the CNS at the perivenular regions, the immune response intensifies, leading to the identification of a broader range of CNS antigens as potential targets for T cells, resulting in a diversification of antigen specificity throughout the disease progression [13]. The main T cell subsets associated with MS are CD8^+^ T cells, CD4^+^ Th1 cells, and Th17 cells. Cytokines such as IFN‐γ, IL‐17, and granulocyte‐macrophage colony‐stimulating factor (GM‐CSF) are produced by autoreactive T cells and may play a role in the pathophysiology of MS [14].

Although MS is predominantly recognized as a T cell‐driven disorder, there is a growing consensus that B cells contribute to its pathogenesis. This is underscored by the common finding of intrathecal immunoglobulin (Ig) production in MS patients, as well as the discovery of antibodies targeting specific myelin antigens associated with the disease [13]. In MS, the majority of B cells in cerebrospinal fluid (CSF) and brain parenchyma are CD27^+^ memory B cells. In MS patients, the rise in intrathecal Ig synthesis is evidenced by multiple factors in the CSF, such as the existence of oligoclonal bands, an elevated immunoglobulin G (IgG) index, B cell clonal expansion, somatic hypermutation, and the B cell receptor editing in expanded B cell clones. Apart from B cell‐related conditions, the loss of proper functioning in effector T cells can impact the course of MS [15].

There are three possible mechanisms contributing to the neurodegenerative process observed in MS. Neurodegeneration signifies the death of nerve cells, which ultimately leads to a total halt in nerve impulse transmission [16]. In MS, this degeneration occurs independently of autoimmune inflammation and is not directly linked to it. MS patients exhibit elevated levels of glutamate in their blood plasma, brain, and CSF. Glutamate acts as an excitatory neurotransmitter in the CNS; however, excessive accumulation can overstimulate N‐methyl‐D‐aspartate (NMDA) receptors on neighboring neurons. This overstimulation results in calcium influx, triggering various pathological processes that eventuate in neuronal death. Therefore, glutamate excitotoxicity is a key mechanism underlying neurodegeneration [16, 17, 18]. An additional mechanism may involve the redistribution of ion channels and changes in their permeability within the axons of neurons. Such changes can disturb the ionic equilibrium, which may ultimately result in axonal damage or even death in extreme situations. Finally, the third mechanism contributing to neurodegeneration could be a change in the balance of remyelination factors that are vital for the survival of oligodendrocytes and neurons in the brain [16].

## 4 Gut Microbiota

In the human intestine, there exists a rich and diverse community of viruses, eukaryotes, archaea, and bacteria, collectively known as the gut microbiota. A significant number of microorganisms inhabit the adult human intestinal tract, with estimates suggesting their total count could reach up to 100 trillion. The gut microbiota is composed of over 1500 species, which are spread across more than 50 distinct phyla. The prevailing phyla include Bacteroidota (formerly known as Bacteroidetes) and Bacillota (formerly known as Firmicutes), which are followed by Actinomycetota (formerly known as Actinobacteria), Pseudomonadota (also known as Proteobacteria), Fusobacteriota, and Verrucomicrobiota [19]. The advent and diversification of the gut microbiota commence at the time of birth, with changes in its makeup reliant predominantly upon various genetic, nutritional, and environmental factors [20]. Actinomycetota, Pseudomonadota, Bacillota, and Bacteroidota are the major phyla that dominate the gut microbiota in infants [21]. The order in which these phyla appear determines their relative abundance. The composition of microbial populations experiences notable shifts during the maturation of individuals, wherein Bacillota takes the lead, followed by Bacteroidota and Actinomycetota [19].

Human health is largely contingent upon the gut microbiota, which functions in a manner reminiscent of a vital internal organ. Under homeostatic conditions, the gut microbiota fulfills a plethora of functions. These include facilitating food digestion, synthesizing vitamins and short‐chain fatty acids (SCFAs), producing neurotransmitters (particularly serotonin), and most importantly, maintaining the integrity of the intestinal barrier and regulating the immune system [22]. The gut microbiota can be shaped by various intrinsic and extrinsic factors, including age, gender, genetic predisposition, personal hygiene practices, dietary behaviors, stress levels, antibiotic consumption, and even exposure to environmental pollutants [23, 24].

It has long been acknowledged that gut microbiota is crucial in fostering the development of the immune system therein, while also occupying a position of prominence in maintaining immune tolerance. This intricate process occurs within the gut‐associated lymphoid tissue (GALT), where specialized cells are adept at discerning between noxious and innocuous antigens. The regulation of GALT by bacteria is facilitated by DCs, which fulfill an antigen‐presenting function. These DCs extend protrusions into the mucous layer on the surface of the intestinal epithelium to interact with bacteria in the intestinal lumen. Following this, DCs display the antigens of the bacteria in the mesenteric lymph nodes to T and B cells. The antigen presentation induces the differentiation of naïve T cells into Tregs, which are specialized in producing anti‐inflammatory cytokines such as IL‐10 and transforming growth factor beta (TGF‐β) [25]. These cytokines are necessary for the maintenance of a tolerogenic environment. IgA antibodies are also synthesized by B cells in response to the presented antigens. Upon translocation, these B cells release IgA antibodies directly into the intestinal lumen [26].

The establishment of a harmonious relationship between the host and gut microbiota is of utmost importance in achieving intestinal homeostasis and effectively thwarting pathogen growth. The presence of dysbiosis signifies an imbalance in microbiota composition, which incites inflammation and undermines the functional capacity of the host′s immune defenses. A consequential outcome of this imbalance is disturbance of intestinal permeability, which eventuates in the emergence of leaky gut and endotoxemia. This, in turn, triggers the initiation and progression of low‐grade systemic inflammation throughout the body, a state that seems to be closely interwoven with the onset of diverse metabolic and autoimmune diseases such as obesity, nonalcoholic fatty liver disease (NAFLD), cardiovascular disease, inflammatory bowel disease, type 1 diabetes mellitus (T1D), and neurodegeneration [27].

## 5 The Gut‐Brain Axis

The gut microbiota and CNS partake in bidirectional communication through a remarkably complex and nuanced system often referred to as the gut‐brain axis. The principal components comprising the axis are the microbiota, the intestinal barrier, the enteric nervous system (ENS), and the vagus nerve [28]. The gut can be influenced by the CNS via direct neural pathways and the hypothalamic‐pituitary‐adrenal axis as part of the stress response. The regulation of various gut functions, including intestinal motility, mucus production, gastric acid secretion, liberation of intestinal peptides, gut permeability, and mucosal immune responses, is under the control of the CNS [29].

**Figure 1:**
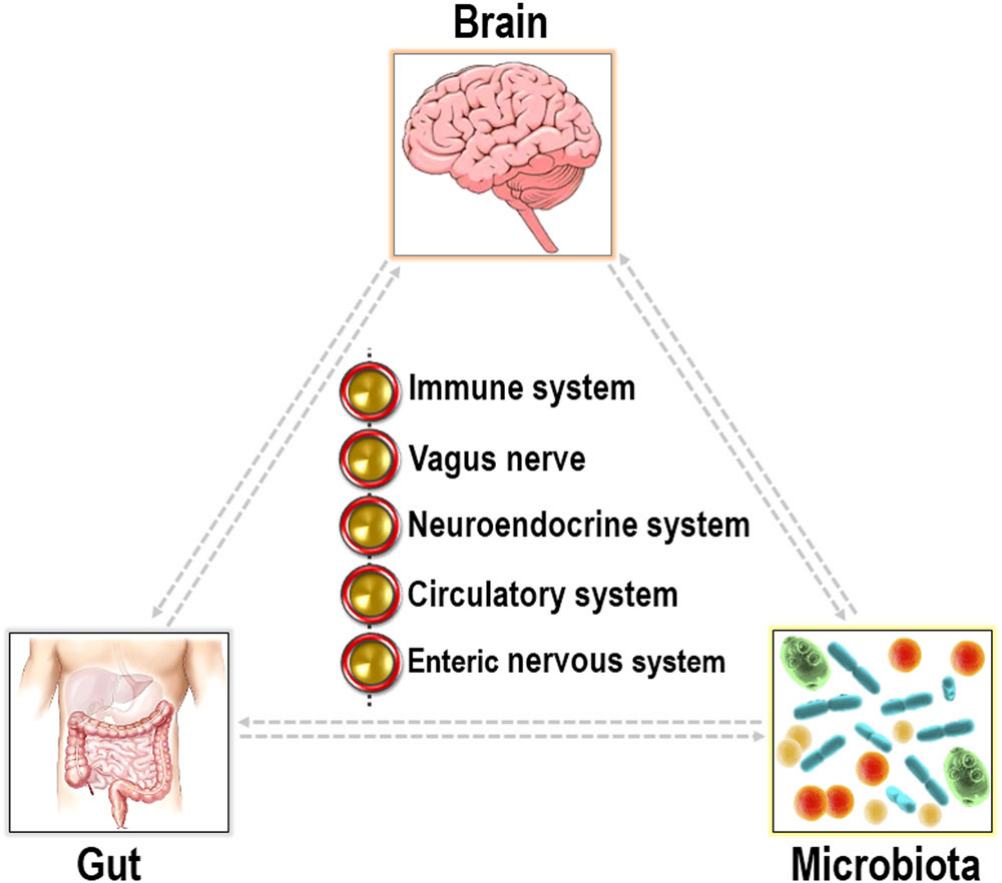
The concept of the gut‐brain axis highlights the bidirectional communication between the brain, gut, and microbiota. This complex network depends upon multiple components, such as the immune system, vagus nerve, neuroendocrine system, circulatory system, and enteric nervous system.

The ENS is the major component of the autonomic nervous system and is equipped with intrinsic microcircuits that empower it to autonomously coordinate gastrointestinal activities without requiring input from the CNS. Hence, it is sometimes called the “second brain” [30]. The ENS is well connected to the CNS via both motor and sensory pathways of the sympathetic (via the prevertebral ganglia) and parasympathetic (via the vagus nerve) nervous systems [31]. The ENS is comprised of a vast array of neurons and enteric glial cells, which are instrumental in preserving the functionality of the intestinal barrier. In a succinct fashion, the ENS presides over several functions, including gastrointestinal motility, secretion, nutrient absorption, immune regulation, and defense [32]. The brain, gut, and intestinal microbiota interact through multiple communication pathways, which are vital for the optimal functioning of the overall system (Figure 1).

The liberation of gut hormones by enteroendocrine cells (EECs), which are sprinkled throughout the gastrointestinal tract, is a noteworthy aspect of the gut‐brain axis since these hormones act as quintessential signaling molecules. Various subtypes of EECs that produce upward of 20 peptides/hormones have been discovered throughout the gastrointestinal mucosa. Salient examples include ghrelin, polypeptide YY (PYY), glucagon‐like peptide‐1 (GLP‐1), glucose‐dependent insulinotropic polypeptide (GIP), cholecystokinin (CCK), and 5‐hydroxytryptamine or serotonin. For instance, serotonin is responsible for influencing intestinal motility, pain perception, electrolyte secretion, cardiac functions, vascular tone, organ development, and inflammation. In addition, it is capable of functioning as a neurotransmitter to regulate mood, sleep, and appetite [33]. Ghrelin is primarily acknowledged for its adipogenic and orexigenic (appetite‐stimulating) properties. It also acts as a regulator of stress response, anxiety, and depression. Ghrelin significantly alleviates excessive inflammation and reduces damage to various target organs, mainly by diminishing the secretion of inflammatory cytokines [34]. GIP and GLP‐1, hormones secreted by the intestine following nutrient intake, occupy a crucial role in stimulating insulin secretion from pancreatic β cells. Moreover, these hormones exert substantial influence upon lipid metabolism, gastric emptying, appetite regulation, and body weight management [35].

**Figure 2:**
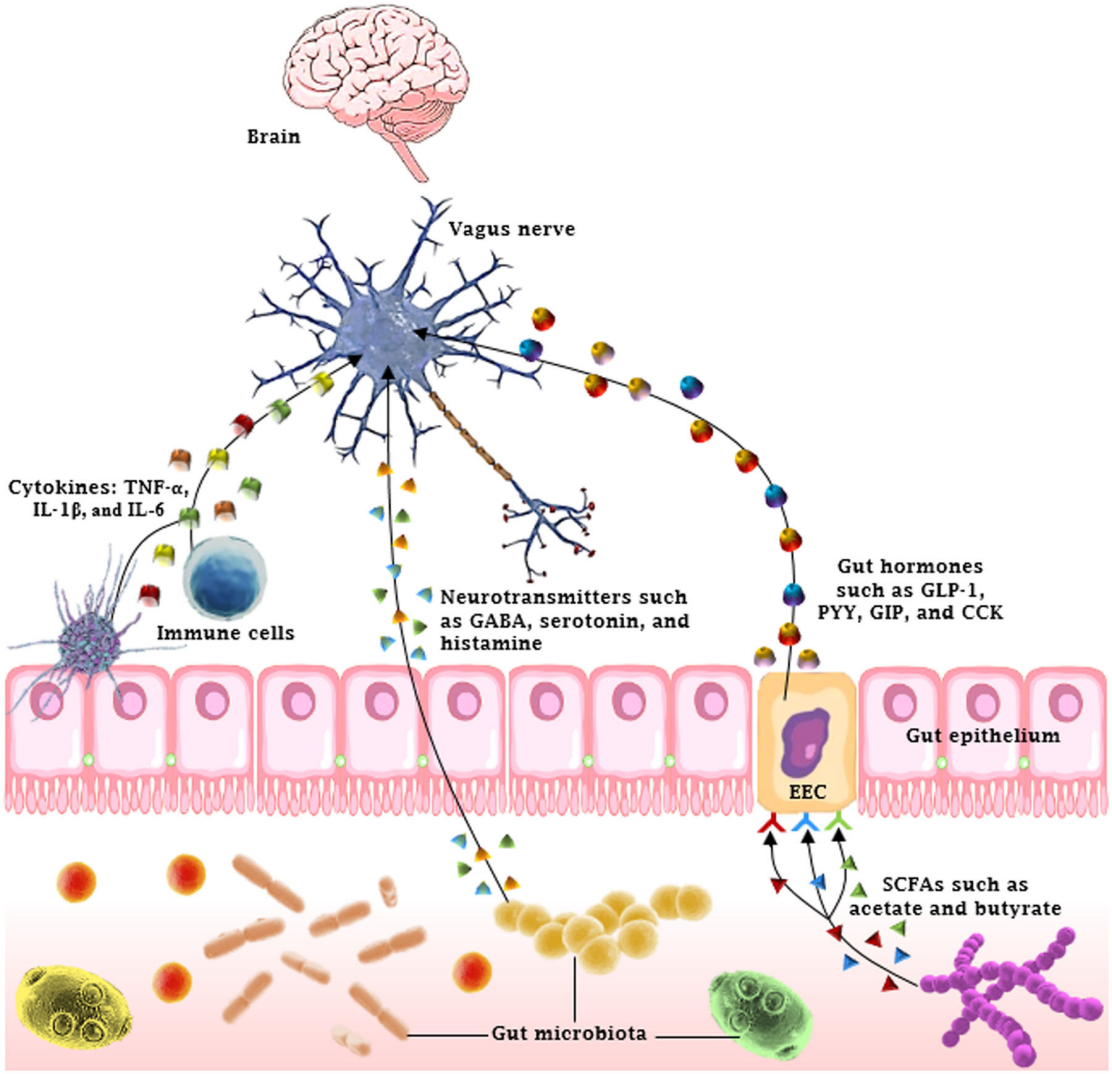
Schematic representation of how the gut microbiota and enteroendocrine cells (EECs) influence the central nervous system through different pathways. CCK, EEC, GABA, GIP, GLP‐1, IL, PYY, SCFAs, and TNF‐α represent cholecystokinin, enteroendocrine cell, γ‐aminobutyric acid, glucose‐dependent insulinotropic polypeptide, glucagon‐like peptide‐1, interleukin, polypeptide YY, short‐chain fatty acids, and tumor necrosis factor alpha, respectively.

The release and function of gut hormones are heavily reliant on the status of the gut microbiome. One must take heed of the fact that gut hormones can affect cognitive functions, like learning and memory, by interacting with different receptors situated in the CNS [33]. Beyond this, a number of bacteria have been demonstrated to produce a range of mammalian neurotransmitters such as dopamine, noradrenaline, serotonin, γ‐aminobutyric acid (GABA), acetylcholine, and histamine [36]. When it comes to metabolites derived from gut microbes, SCFAs such as butyrate, propionate, and acetate are currently the focus of research related to inflammatory diseases (Figure 2). They are key candidates involved in the crosstalk between gut microbes and host cells. The influence of SCFAs extends to multiple host physiological processes, encompassing gastrointestinal function, blood pressure control, circadian rhythms, and immunomodulation [37, 38]. Neurological and psychiatric disorders such as anorexia nervosa, PD, AD, and chronic stress have all shown a decrease in SCFAs levels [39]. Conversely, elevated levels have been recorded in obesity and ASD [40].

## 6 Changes of Gut Microbiota in MS Patients

Analysis of fecal microbiota in MS patients has brought to light a discernible imbalance in certain bacteria, indicating both depletion and enrichment when juxtaposed with healthy controls. As for phyla, there is a discrepancy in findings associated with Bacillota [41, 42, 43, 44, 45, 46] and Actinomycetota [42, 47] in several studies. Pseudomonadota and Lentisphaerota also demonstrated a decline among MS patients as opposed to the healthy controls [42]. Evidence from various studies points to an increase in Bacteroidota in MS patients [47, 48, 49], but one study contradicted this by showing a decrease [41]. One study also reported an increase in the population of Verrucomicrobiota in MS patients [50]. Regarding families, a reduction in Lachnospiraceae was noted in two studies [41, 44], while other studies have reported mixed findings on Ruminococcaceae [47, 50].

Upon conducting a genus‐level analysis, researchers have noted a decrease in the population of *Bifidobacterium* [41, 47], *Roseburia* [43, 44, 46], *Coprococcus* [44, 49], *Butyricicoccus* [43, 46], and *Lachnospira* [41, 44], *Haemophilus* [43] among MS patients compared with healthy controls. In this context, members of the genus *Bifidobacterium* are believed to bestow their protective benefits via an array of mechanisms, including the production of SCFAs, changes in lipid profile, induction of Treg differentiation, and modulation of Th1/Th2 balance [51]. The reduced abundance of *Roseburia* has also been documented across a multitude of disorders such as juvenile idiopathic arthritis, Behcet′s syndrome, irritable bowel syndrome, obesity, type 2 diabetes, metabolic syndrome, liver cirrhosis, hepatic encephalopathy, cholelithiasis, PD, and allergies [52]. *Roseburia*, *Coprococcus*, *Butyricicoccus*, and *Lachnospira* are all proficient at producing SCFAs, particularly butyrate, and have been associated with various neurological diseases [53]. Patients on disease‐modifying treatment show increased abundances of *Sutterella* population in their gastrointestinal tract [54]. *Sutterella* is a Gram‐negative bacterium known for its capability to degrade IgA in the gut. Interestingly, some studies have shown that gut microbiota‐specific IgA cells could serve as systemic mediators in MS, underscoring the critical function of mucosal B cells in active neuroinflammation. This raises the possibility of using IgA as a new biomarker and targeting IgA‐producing cells as an immune subset for therapeutic purposes [55, 56].

**Figure 3:**
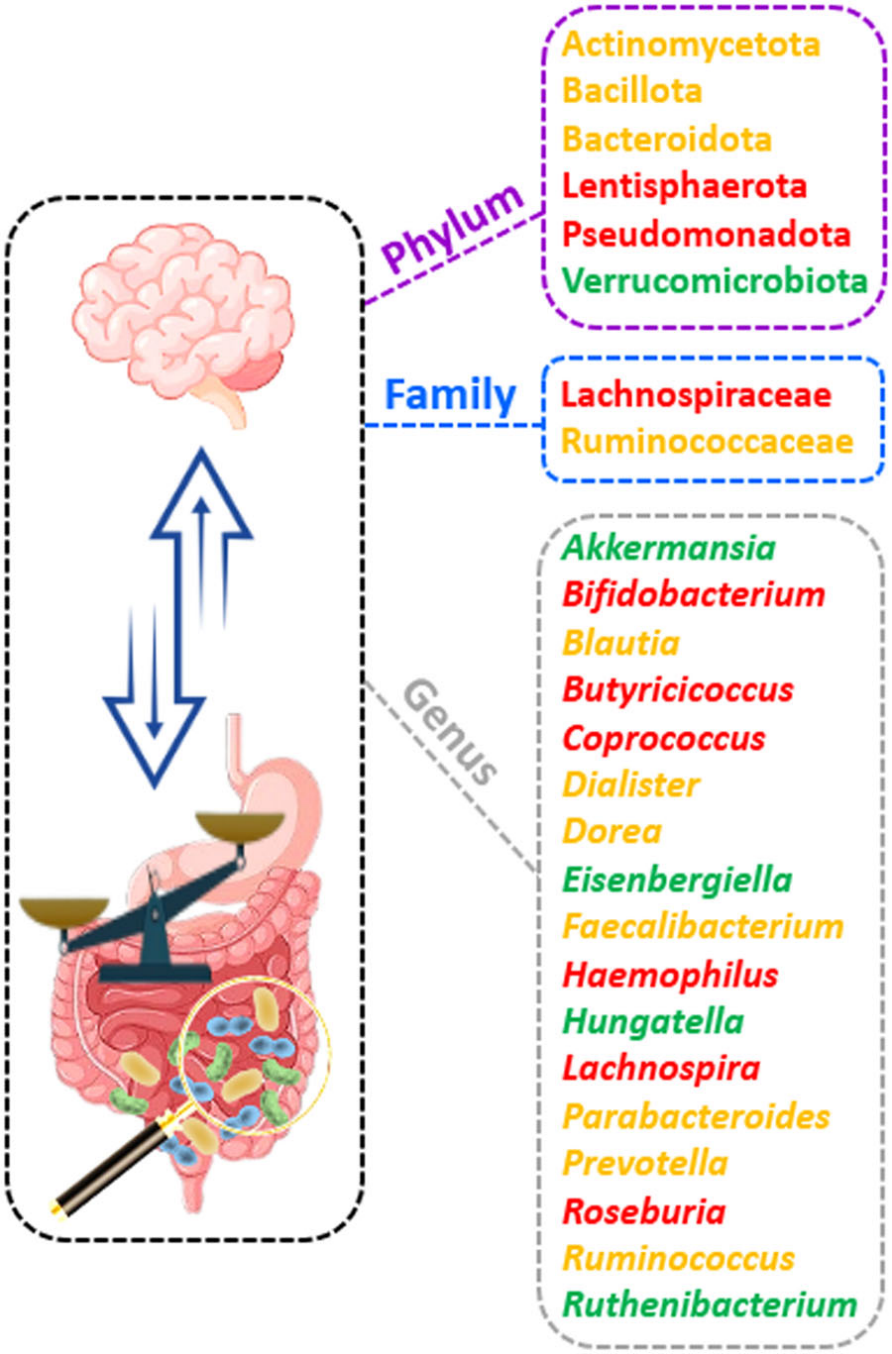
Changes in the populations of phyla, families, and genera of gut microbiota in MS patients. Green represents an increase in population for family, genus, and phylum, red indicates a decrease, and orange denotes inconsistent outcomes in relevant studies.

On the other hand, some studies have disclosed a rise in the populations of *Akkermansia* [45, 50, 57]. A recent study also pointed out an increase in the populations of the genera *Ruthenibacterium*, *Hungatella*, and *Eisenbergiella* [57]. Other investigations indicated inconsistent results related to the population shifts of genera such as *Blautia* [42, 43, 44, 45, 47, 57], *Parabacteroides* [41, 43, 46, 47], *Dorea* [43, 44, 45], *Ruminococcus* [42, 44, 46], *Faecalibacterium* [41, 42, 43, 57], *Prevotella* [41, 44, 45], and *Dialister* [42, 45]. Among these various genera, *Akkermansia* stands out as a notable exemplar, especially when considering its importance to MS. *Akkermansia* exhibits immunoregulatory properties through the conversion of mucin into SCFAs. Nevertheless, the breakdown of the intestinal mucosa by *Akkermansia* may eventuate in intestinal inflammation. This mechanism contributes to the impairment of the intestinal barrier, thereby promoting an increased presentation of microbial antigens to the resident immune cells. Furthermore, the inflammatory effects of *Akkermansia* could potentially be associated with the increased expression of genes responsible for antigen presentation as well as the initiation of the complement cascade [58]. Figure 3 depicts the changes in the populations of phyla, families, and genera of gut bacteria among MS patients, according to the consensus findings.

An additional aspect worthy of mention is the impact of MS treatment on gut dysbiosis. Disease‐modifying therapies (DMTs) refer to particular immunomodulatory agents that are intended to lower disease activity as observed clinically and through radiological assessments [59]. These drugs are sorted into two groups based on their efficacy: moderate‐efficacy DMTs, which include interferon beta (IFN‐β), teriflunomide (TER), glatiramer acetate (GA), and dimethyl fumarate (DMF), and high‐efficacy DMTs, which feature natalizumab, anti‐CD20 antibodies, cladribine, and sphingosine 1‐phosphate receptor modulators [60].

For instance, in a study conducted on 50 MS patients and 21 healthy controls, 20 patients received DMTs (IFN‐β1 or TER), 19 patients received DMTs in conjunction with homeopathy, while 11 patients accepted solely homeopathy. In comparison to healthy controls, untreated MS patients exhibited a reduction of Actinomycetota, *Bifidobacterium*, *Faecalibacterium prausnitzii*, and an increase of *Prevotella stercorea*, whereas treated patients demonstrated diminished *Ruminococcus* and *Clostridium*. When analyzing the treated MS group relative to their initial state, there was a decrease in Lachnospiraceae and *Ruminococcus* and an increase in *Enterococcus faecalis*. In addition, *Eubacterium oxidoreducens* levels were lower following homeopathic treatment. The administration of IFN‐β1, TER, or homeopathy revealed several notable taxonomic alterations. The authors concluded that DMTs and homeopathy may exert an influence on the gut microbiota [61]. In a similar vein, another recent investigation examined the shifts in gut microbiota among 60 subjects, comprising 39 MS patients and 21 healthy controls, both before and after undergoing a DMT, which included either IFN‐β1 or TER. In addition, 19 participants were treated with a combination of traditional DMTs alongside an immunoglobulin Y (IgY) supplement. When compared to the healthy control group, those with MS displayed an increase in *Prevotella stercorea* and a reduction in *Faecalibacterium prausnitzii*. Following treatment, MS patients experienced increased levels of Lachnospiraceae and *Streptococcus*. The second set of samples indicated a rise in *Bifidobacterium angulatum* and a decline in *Oscillospira* among the MS participants. This study underscored the potential for DMTs and IgY supplements to influence microbial composition, which may help in restoring a more balanced microbiome in affected patients [62].

In a different recent investigation, the gut microbiota of 19 patients with MS was examined at baseline and subsequently at 1, 3, and 6 months following DMF treatment. At the genus level, a reduction in *Clostridium* abundance was noted after 6 months of DMF. In those patients experiencing side effects, a higher prevalence of *Streptococcus*, *Haemophilus*, *Clostridium*, *Lachnospira*, *Blautia*, *Subdoligranulum*, and the phylum Mycoplasmatota was observed, alongside a decrease in *Barnesiella*, *Odoribacter*, *Akkermansia*, and the phyla Pseudomonadota and Bacteroidota [63]. In a separate study, the impact of treatment‐naïve patients or those treated with GA or DMF on gut microbiota alterations in relapsing MS was examined. The use of DMTs correlated with modifications in the composition of fecal microbiota. Both treatment options were linked to a reduction in the relative abundance of the Lachnospiraceae and Veillonellaceae families. Furthermore, DMF was associated with a decline in the relative abundance of the phyla Bacillota and Fusobacteriota, as well as the order Clostridiales, while showing an increase in the phylum Bacteroidota. This study underscored that the administration of GA or DMF leads to shifts in gut microbial composition among MS patients [64].

Overall, DMTs used to treat MS have been shown to significantly affect the gut‐brain axis. Given that the gut microbiota influences both systemic and local immune responses, the alterations in commensal microbiota composition that DMTs cause may be linked to varying levels of treatment success and negative effects experienced by MS patients. In addition, a patient′s gut microbiota may serve as a biomarker for a favorable reaction to a particular DMT, which may aid in selecting the optimal immunomodulatory therapy. Validation of these theories through upcoming research could be crucial for personalized medicine [59]. Furthermore, it is essential to conduct future randomized controlled trials involving a broader population to improve our comprehension of how DMTs influence the makeup of gut microbiota, as the current research is constrained by small sample sizes. Furthermore, the implementation of uniform protocols for human fecal sample collection could serve as a valuable strategy for obtaining more credible results. It has also been observed that numerous studies have neglected to compare long‐term follow‐ups with single DMT alone. Conducting long‐term evaluations would enable comparisons among patients exhibiting similar clinical courses, thereby facilitating a more comprehensive understanding of the gut microbiota′s role in the pathophysiology of relapse and the effectiveness of DMTs.

## 7 Targeting Gut Microbiota for the Treatment of MS

**Figure 4:**
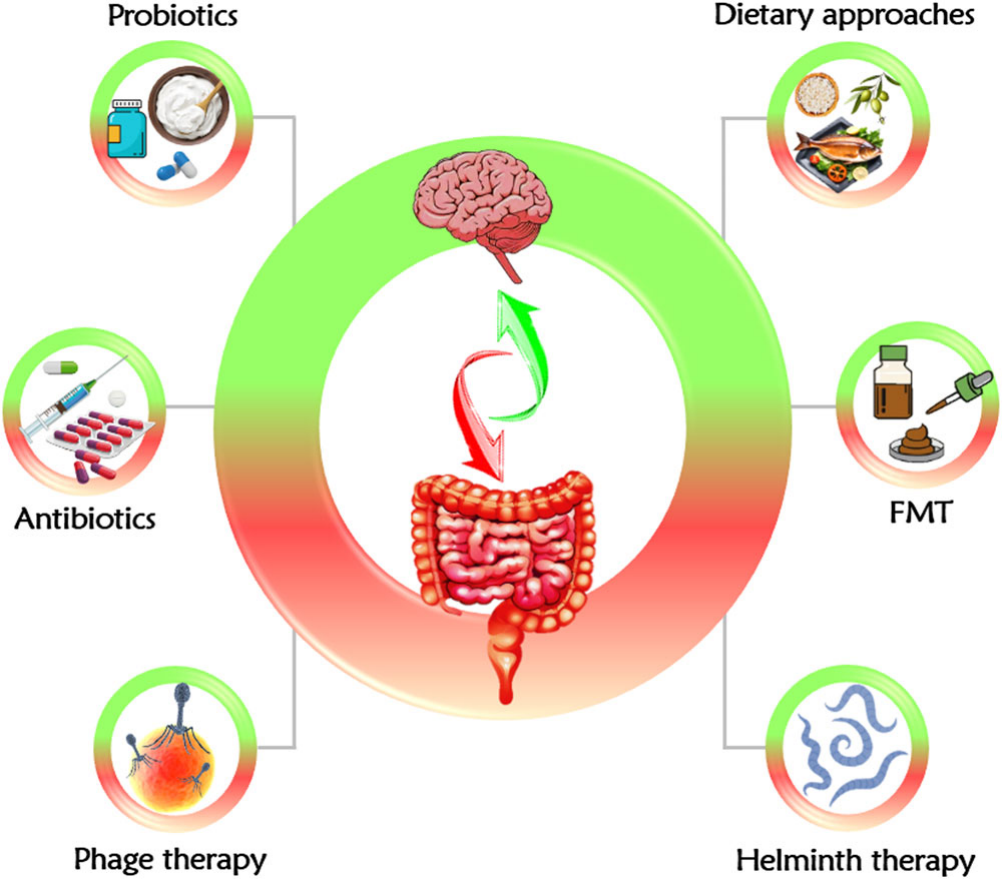
A schematic overview of different potential therapeutic interventions for MS treatment, encompassing antibiotics, probiotics, dietary approaches, fecal microbiota transplantation (FMT), helminth therapy, and phage therapy.

Considering the crucial function of gut microbiota in regulating immune responses and the importance of dysbiosis in neuroinflammatory conditions, therapeutic approaches to restore gut microbiota balance could offer valuable avenues for managing MS. Despite the absence of an approved MS treatment focusing on the gut microbiota, it is imperative to undertake a scrupulous examination of the proposed therapeutics while giving precedence to the development of treatments with minimal adverse effects. In what follows, we shall touch upon various strategies geared toward targeting the gut microbiota for the treatment of MS (Figure 4).

### 7.1 Antibiotics and Other Antimicrobial Agents

The two‐sided nature of antibiotics is evident in their capacity to eliminate harmful bacteria while also disturbing the natural harmony of beneficial microbes in the gut, ultimately leading to dysbiosis. The effects of administering antibiotics on the gut microbiota are contingent upon the type of antibiotics prescribed and the duration of the treatment regimen. Even though the utilization of antimicrobials in animal models yielded favorable outcomes, it is noteworthy that only minocycline and hydroxychloroquine showed improvements in outcome measures for patients with MS [65].

Minocycline has already exhibited neuroprotective effects in several neurodegenerative disorders, such as PD, HD, amyotrophic lateral sclerosis, and stroke [66, 67]. The anti‐inflammatory effect of minocycline in MS is achieved through its ability to inhibit T cell migration and the release of matrix metalloproteinases that can disrupt the integrity of the BBB [68]. In regard to hydroxychloroquine, positive outcomes were observed in an experimental autoimmune encephalomyelitis (EAE) animal model and in a clinical trial, leading to the mitigation of the severity of the disease [69, 70]. In view of the connection between certain viruses (such as EBV, cytomegalovirus, and varicella zoster virus) and MS, researchers have sought to unravel the potential of antiviral medications, including acyclovir, valacyclovir, and ganciclovir, in treating the disease [65, 71]. Unfortunately, human trials have not shown promising results as of yet [65]. From what has been discussed above, it seems advisable to initiate comprehensive, multicenter, and well‐designed studies to probe more deeply into the impacts of antimicrobials on MS patients.

### 7.2 Probiotics, Prebiotics, Postbiotics, and Synbiotics

The current definition of “probiotics” was established in 2001 by the Food and Agriculture Organization of the United Nations and the World Health Organization (FAO/WHO), which describes them as “live microorganisms that, when given in sufficient quantities, provide health benefits to the host”. This definition has since become the most widely recognized and accepted globally [72, 73]. Psychiatrist Ted Dinan and neuroscientist John F. Cryan coined the term “psychobiotics” to describe probiotics that support the mental health of patients with mental illnesses. The efficacy of these psychobiotics is related to their effects on the gut‐brain axis [74]. Psychobiotics encompass probiotics, prebiotics, synbiotics, and postbiotics that are utilized to tackle mental health issues by offering various benefits. Beyond their favorable psychological impacts, psychobiotics can promote the release of neurotransmitters and neurohormones that can produce psychotropic effects [75]. The common psychobiotic bacteria belong to the genera *Lactobacillus*, *Streptococcus*, *Bifidobacterium*, *Escherichia*, and *Enterococcus* [76].

Prebiotics refer to non‐digestible substances in food that promote health by altering the microbiota in the host [77]. Prebiotics can be utilized either as a replacement for probiotics or as an additional aid to their function. Nonetheless, it is crucial to understand that different prebiotics can stimulate the growth of specific indigenous gut bacteria. Prebiotics exist in different forms, with inulin, fructooligosaccharides (FOS), and galactooligosaccharides (GOS) being the most frequently used by individuals [78]. On the other hand, the term “synbiotics” refers to formulations that integrate prebiotics and probiotics in a complementary way. This combination is intended to increase the survival rates of probiotic microorganisms in the digestive tract and to foster the growth of particular native microbial populations. Commonly used formulations in synbiotic products usually involve a combination of FOS with various species from the genera *Bifidobacterium* or *Lactobacillus* [79]. The International Scientific Association for Probiotics and Prebiotics (ISAPP) describes a postbiotic as a “preparation of inanimate microorganisms and/or their components that confers a health benefit on the host” [80].

A considerable wave of curiosity has lately emerged regarding the conceivable therapeutic advantages that probiotics may offer in the realm of neurological disorders. *Lactobacillus*, *Lactococcus*, and *Bifidobacterium* are the most common types of probiotics, recognized as safe by the US Food and Drug Administration (FDA). They are found in various forms, such as capsules, tablets, sachets, and fermented milk products [81]. Research has brought to the forefront the importance of probiotics in providing neuroprotection and effectively managing neurological conditions, including ASD, PD, and AD. Convincing findings also lend credence to the idea that probiotics can wield a favorable influence upon mood states, such as reducing anxiety, stress, and depression. In addition, they have demonstrated potential in improving cognitive impairment and aiding in the recovery of CNS injuries [82].

The key findings gleaned from studies exploring the effects of probiotics, prebiotics, synbiotics, and postbiotics upon individuals with MS are outlined in Table 1. Despite the uncertainty surrounding the exact mode of action of probiotics, it is probable that several mechanisms are working in tandem. Probiotics exhibit a wide array of functionalities, such as supporting the maintenance of the gastrointestinal barrier, regulating the balance of intestinal microbes, thwarting the colonization of opportunistic pathogens, preventing the adhesion of gut pathogens, inhibiting their growth via the secretion of compounds like lactic acid, propionic acid, acetic acid, bacteriocins, and reactive oxygen species, and modulating both the innate and acquired immune responses [83, 84].

**Table 1:** Summary of the most relevant clinical studies investigating the efficacy of probiotics, prebiotics, synbiotics, and postbiotics supplementation in MS patients.

Study group	Country	Intervention	Treatment duration	Key findings	References
RRMS patients treated with GA (*n* = 7) or untreated (*n* = 2) and healthy controls (*n* = 13)	The USA	Oral administration of VSL#3 (containing 36 × 10^11^ CFUs/day of *Bifidobacterium breve, Bifidobacterium longum subsp. infantis, Bifidobacterium longum* subsp. *longum*, *Lacticaseibacillus casei, Lactiplantibacillus plantarum, Lactobacillus acidophilus, Lactobacillus delbrueckii* subsp. *bulgaricus*, and *Streptococcus salivarius* subsp. *thermophilus*) twice daily	2 months	↑*Lactobacillus* in the VSL#3‐treated patients ↓*Akkermansia* and ↓*Blautia* in the VSL#3‐treated patients ↓The abundance of several KEGG pathways associated with altered gut microbiota function in RRMS patients, such as methane metabolism No serious adverse reactions or relapse in RRMS patients	[89]
RRMS patients treated with GA (*n* = 7) or untreated (*n* = 2) and healthy controls (*n* = 13)	The USA	Oral administration of VSL#3 (containing 36 × 10^11^ CFUs/day of *Bifidobacterium breve, Bifidobacterium longum subsp. infantis, Bifidobacterium longum* subsp. *longum*, *Lacticaseibacillus casei, Lactiplantibacillus plantarum, Lactobacillus acidophilus, Lactobacillus delbrueckii* subsp. *bulgaricus*, and *Streptococcus salivarius* subsp. *thermophilus*) twice daily	2 months	Induction of an anti‐inflammatory peripheral immune response characterized by ↓frequency of intermediate monocytes (CD14^high^CD16^low^) in RRMS patients following VSL#3 administration ↓MFI of HLA‐DR on DCs in RRMS patients following VSL#3 administration ↓MFI of CD80 on classical monocytes in healthy controls following VSL#3 administration ↓Frequency of inflammatory monocytes in healthy controls following VSL#3 administration	[90]
RRMS patients (*n* = 48), including the probiotic group (*n* = 24) and the placebo group (*n* = 24)	Iran	Oral administration of a probiotic capsule (containing 2 × 10^9^ CFUs/day of *Bifidobacterium animalis* subsp. *lactis, Bifidobacterium longum* subsp. *infantis*, *Lacticaseibacillus casei, Lactiplantibacillus plantarum, Limosilactobacillus fermentum*, and *Limosilactobacillus reuteri*) and a placebo capsule (containing maltodextrin) to the probiotic group and the placebo group, respectively	16 weeks	↓EDSS, ↓BDI, ↓GHQ, ↓DASS, ↓MDA, ↓8‐OHdG, ↓IL‐6, ↓hsCRP, ↑IL‐10, and ↑NO levels in the probiotic group compared with the placebo group No significant effects on SOD, GSH, TAC, TNF‐α, FPG and QUICKI	[91]
RRMS patients (*n* = 65), including the probiotic group (*n* = 32) and the placebo group (*n* = 33)	Iran	Daily oral administration of two probiotic capsules (containing 2×10^9^ CFUs/capsule of *Bacillus subtilis*, *Bifidobacterium bifidum*, *Bifidobacterium breve*, *Bifidobacterium longum* subsp. *infantis*, *Bifidobacterium longum* subsp. *longum, Lacticaseibacillus casei, Lacticaseibacillus rhamnosus, Lactiplantibacillus plantarum, Lactobacillus acidophilus*, *Lactobacillus delbrueckii* subsp. *bulgaricus*, *Lactobacillus helveticus, Lactococcus lactis* subsp. *Lactis, Ligilactobacillus salivarius*, and *Streptococcus thermophilus*) and two placebo capsules (containing maltodextrin) to the probiotic group and the placebo group, respectively	6 months	↑BDNF, ↓IL‐6, ↓GHQ, ↓BDI, ↓FSS, and ↓PRI in the probiotic group compared with the placebo group	[92]
RRMS patients (*n* = 40), including the probiotic group (*n* = 20) and the placebo group (*n* = 20)	Iran	Daily oral administration of a probiotic capsule (containing 10^10^ CFUs of *Saccharomyces boulardii*, a lactose filler, and a magnesium acetate oil) and a placebo capsule (an identical capsule devoid of any microorganisms) to the probiotic group and the placebo group, respectively	4 months	↑TAC, ↓hsCRP, ↓pain intensity (measured by VAS), and ↓fatigue severity (measured by FSS) in the probiotic group compared with the placebo group ↓MDA in both the probiotic and placebo groups ↑Quality of life in the probiotic group	[93]
PPMS, SPMS, and PRMS patients (*n* = 69), including synbiotic‐diet group (*n* = 34) and the placebo group (*n* = 35)	Iran	Daily oral administration of a synbiotic capsule (containing 4.5 × 10^11^ CFUs of *Bifidobacterium breve*, *Bifidobacterium longum* subsp. *infantis*, *Bifidobacterium longum* subsp. *longum*, *Lacticaseibacillus casei*, *Lactiplantibacillus plantarum, Lactobacillus acidophilus*, *Lactobacillus delbrueckii* subsp. *bulgaricus*, and *Streptococcus thermophilus*, as well as 100 mg of FOS) together with an AI‐AO‐rich diet and a placebo capsule (containing starch) to the synbiotic/diet group and the placebo group, respectively	4 months	A high adherence rate (98%) for the synbiotic/diet Improvements in fatigue, pain, sexual function, and bowel/bladder status in the synbiotic‐diet group compared with the placebo group No serious adverse effects in the synbiotic/diet group	[94]
RRMS patients receiving anti‐CD20 therapy including group 1 (*n* = 22) and group 2 (*n* = 15)	The USA	Group 1: Patients enrolled in the original cross‐over study where probiotic (*Visbiome*, containing 36 × 10^11^ CFUs of *Lactobacillus*, *Bifidobacterium*, and *Streptococcus* species per packet) or prebiotic (*Prebiotin*, containing 4000 mg of OEI per packet) supplementation for 6 weeks was randomized, each followed by a washout period (6 weeks). Group 2: Patients received only prebiotics for 6 weeks followed by an 18‐week washout period. Patients took 2 packets twice daily (4 packets total per day) of either *Visbiome* or *Prebiotin*.	6 weeks	Comparable adherence rates for prebiotics and probiotics in RRMS patients Substantial improvement in patient‐reported bowel control (but not other PROs like disability, fatigue, and mood) with probiotic supplementation No serious adverse effects in RRMS patients receiving the prebiotic and probiotic supplements	[95]

Abbreviations: 8‐OHdG, 8‐hydroxy‐2′‐deoxyguanosine; ↑, increased; ↓, decreased; AI‐AO, anti‐inflammatory‐antioxidant; BDI, Beck depression inventory; BDNF, brain‐derived neurotrophic factor; CFUs, Colony forming units; DASS, depression anxiety and stress scale; DCs, dendritic cells; EDSS, expanded disability status scale; FOS, fructooligosaccharides; FPG, fasting plasma glucose; FSS, fatigue severity scale; GA, glatiramer acetate; GHQ, General health questionnaire; GSH, glutathione; HLA, human leukocyte antigen; hsCRP, high‐sensitivity C‐reactive protein; IL, interleukin; KEGG, Kyoto Encyclopedia of Genes and Genomes; MDA, malondialdehyde; MFI, mean fluorescence intensity; MS, multiple sclerosis; NO, nitric oxide; OEI, oligofructose‐enriched inulin; PPMS, primary progressive MS; PRI, pain rating index; PRMS, progressive relapsing MS; PROs, patient‐reported outcomes; QUICKI, quantitative insulin sensitivity check index; RRMS, relapsing‐remitting multiple sclerosis; SOD, superoxide dismutase; SPMS, secondary progressive MS; TAC, total antioxidant capacity; The USA, The United States of America; VAS, visual analog scale.

Genetically engineered probiotics present another intriguing avenue of exploration, with possible implications for future studies focused on the treatment of MS. The anti‐inflammatory cytokine IL‐10 is crucial for inhibiting inflammatory cascades and is a prime candidate for probiotic engineering [85]. When administered in the dextran sodium sulfate (DSS)‐induced mouse colitis model, a *Lactococcus lactis* strain expressing murine IL‐10 showed both preventive and therapeutic properties [86]. In EAE models, the use of genetically engineered probiotics expressing heat shock protein and elafin (an endopeptidase that prevents elastase‐mediated tissue proteolysis associated with inflammatory bowel disease, IBD) has exhibited promising anti‐inflammatory effects [87, 88]. The expression of IL‐10, IL‐35, and metabolites such as SCFAs, neurotransmitters, and gut hormones in genetically engineered probiotics may present a hopeful path for the management of MS [85].

### 7.3 Dietary Approaches

The human diet significantly influences the composition of the gut microbiota. Verily, the configuration of the gut microbiota is primarily dictated by the dietary preferences of an individual, since they impact the availability of nutrients for the microbial inhabitants. Subsequently, this engenders fluctuations in key metabolites that affect the immune system. Managing MS patients through dietary interventions often involves the following popular dietary regimens: the Mediterranean, the Paleolithic, the Swank, the McDougall, and the caloric restriction diets [96, 97]. Regardless of the positive health outcomes tied to specific dietary changes, it ought to be noted that the majority of these diets may not be sustainable over the long haul and could culminate in nutrient deficiencies [62].

Extensive research has hitherto been dedicated to exploring the effects of the Mediterranean diet on alleviating the severity of neuroinflammation in MS patients [98]. Prioritizing the consumption of plant‐based foods, the Mediterranean diet encourages a high intake of fruits, vegetables, nuts, legumes, grains, and olive oil. When following the Mediterranean diet, it is recommended to consume fish and poultry in low to moderate amounts, while abstaining from red meat and processed foods altogether. Evidence suggests that adherence to the diet can impact the gut microbiota and alleviate inflammation, leading to improvements in symptoms such as fatigue, quality of life, relapse rate, and cognitive impairment in MS patients [99]. By affecting brain‐derived neurotrophic factor (BDNF) levels, this dietary intervention boosts cognitive function in a favorable way. The restoration of myelin owes much to the instrumental role of BDNF, as it actively participates in its repair. Additionally, BDNF is essential not only for neurodevelopment but also for the function and survival of neurons in the adult brain [100].

The global movement toward adopting vegan and vegetarian diets has attracted attention for its ethical implications, environmental sustainability, and potential health benefits. These diets are often rich in phytonutrients and antioxidants, which have been associated with lower inflammatory markers, including C‐reactive protein (CRP) and IL‐6, indicating a possible protective effect against systemic inflammation and oxidative stress. Nonetheless, even with these advantages, there are ongoing concerns about their effects on neurological health, particularly owing to potential deficiencies in essential nutrients such as vitamin B12, docosahexaenoic acid (DHA), eicosapentaenoic acid (EPA), and iron [101]. The McDougall diet is a prominent example of a plant‐based diet, characterized as a vegan regimen that is extremely low in fat and primarily relies on complex carbohydrates for the majority of its caloric intake. It eliminates all types of meat and animal‐based fats, including fish, dairy, and eggs. Evidence suggests that MS patients who adhered to the McDougall diet experienced lower levels of fatigue. However, the diet did not demonstrate a notable impact on relapse rates, magnetic resonance imaging (MRI) activity, or overall disability [102]. According to a new systematic review of clinical trials, a plant‐based diet is considered essential for dietary recommendations for MS patients. Nevertheless, the review highlights that low‐fat, low‐calorie, and ketogenic diets, especially when enhanced with fish oil, vegetable oil, and flavonoids, could also be advantageous [103]. On the other hand, MS is commonly associated with a range of mental disorders, including depression, anxiety, and various mood or emotional disturbances. These issues can be directly linked to the neurological damage caused by MS, but they may also be affected by psychosocial factors. Addressing these mental health issues is crucial for enhancing the overall well‐being and quality of life for MS patients [104]. Because of this, the relationship between diets, the influence of the gut microbiota, and mental disorders in MS patients should be highlighted in future studies. In the future, nutritional psychiatry could have significant implications, including the application of diagnostic testing of the gut microbiome to discover targets for personalized psychological and psychiatric care, along with the potential for integrative methods that combine dietary modifications, medication, and cognitive‐behavioral therapy [105].

Evidence amassed from randomized clinical trials (RCTs) also attests to the advantageous effects of particular compounds such as melatonin, vitamin D3, omega‐3 polyunsaturated fatty acids, and polyphenols in the management of MS [106]. For example, vitamin D levels are inversely correlated with disability scores, but did not yield any noteworthy outcomes in terms of relapses, MRI findings, and disability progression [107]. In addition, there is evidence supporting a decrease in disability scores with the administration of high‐dose biotin [108, 109]. The presence of a negative correlation between the serum concentration of vitamin B12 and disability scores has also been documented [110]. Overall, uncertainty arises due to the methodological limitations of RCTs in this area, while observational studies lack a prospective longitudinal design and valid outcome measures. Such constraints emphasize the critical need for future research studies that adhere to high‐quality standards.

### 7.4 Fecal Microbiota Transplantation (FMT)

The principal aim of FMT is to rectify dysbiosis in the recipients by the reconstruction of their intestinal microflora using microbiota from healthy donors. The effectiveness of FMT is remarkably high, rendering it an approved therapeutic intervention for managing patients with recurrent and refractory *Clostridium difficile* infections [111]. Beyond its intended use in treating gastrointestinal disorders, FMT has proven to be advantageous in tackling neurological and psychological symptoms by regulating the gut‐brain axis. FMT has lately gained momentum as a treatment option for neurological and psychiatric disorders, including PD, AD, epilepsy, Tourette′s syndrome, chronic fatigue syndrome, ASD, bipolar disorder, depression, hepatic encephalopathy, and neuropathic pain [111, 112]. A recent review also cast light upon the positive impact of FMT on motor symptoms, mobility, and gastrointestinal function in MS patients. Importantly, these improvements were sustained during follow‐up years, with no adverse events noted [113]. A possible explanation for the positive effects of FMT administration is a decrease in serum concentration of inflammatory markers like IL‐6, IL‐8, and TNF‐α, alongside an increase in beneficial stool bacteria (notable changes in the relative abundances of butyrate‐producing bacterial species *Faecalibacterium prausnitzii*, *Eubacterium rectale*, and *Collinsella aerofaciens*), SCFAs, and serum BDNF levels [114]. Various factors, including the method of transplantation, the origin of donors, and the use of antibiotics prior to the procedure, can potentially influence the effectiveness of FMT [115]. To pave the way for the widespread adoption of this novel technique for MS treatment, it is crucial to implement multi‐center RCTs that further evaluate and strengthen these results.

### 7.5 Phage Therapy

The human virome is dominated by phages, accounting for roughly 90% of its composition. These viruses are tailored to attack specific bacteria, allowing for precision in targeting desired populations while leaving others unscathed. The indubitable contribution of phages to the eubiosis of the human microbiota cannot be downplayed [116]. Phage therapy has a long‐standing history in Eastern Europe, dating back to the early 1900s. However, owing to a meager grasp of phage biology during its early days, coupled with exaggerated claims and a lack of controlled trials, phage therapy gradually receded into the background as antibiotics gained prominence [117].

Notwithstanding the exiguous clinical evidence affirming the efficacy of phage therapy, several studies have illustrated the ability of phages to modify the gut microbiota. With regard to the gut microbiota, phages have proven to be effective in eliminating adherent‐invasive *Escherichia coli* in a mouse model of colitis and in patients with Crohn′s disease in remission [118, 119]. Although phages hold potential, they may be harmful by contributing to intestinal dysbiosis. The uncontrolled elimination of beneficial bacteria by phages may disrupt the balance and functionality of the gut microbiota. Beyond this, phages can inadvertently pass on the antibiotic resistance genes to their bacterial hosts through horizontal gene transfer mechanisms. Researchers are actively pursuing the development of smart or engineered phages that may be altered to broaden their host range, prevent lysogeny, and avoid horizontal gene transfer [120]. There remain numerous unresolved queries and worries surrounding the safety and efficacy of phage therapy, especially pertaining to its impact on the gut microbiota for the treatment of MS.

### 7.6 Helminth Therapy

Over the past years, the hygiene hypothesis, also known as the “old friends” hypothesis, has come into the limelight. The hypothesis propounds a link between the simultaneous reduction in exposure to microbes and helminths as a result of improved sanitation and vaccination, and the rise of autoimmune diseases in developed nations [121]. Helminths are key regulators of inflammatory reactions, as the protective immune response to helminth infections involves the activation of Th2 cells and the production of anti‐inflammatory cytokines such as IL‐4, IL‐5, IL‐9, and IL‐13. Moreover, the colonization of helminths in the intestines brings about changes in the composition of the gut microbiota, ultimately leading to the suppression of intestinal inflammation. Accordingly, the possible implementation of helminth therapy in the management of various autoimmune diseases is a topic that has piqued the interest of numerous researchers [122].

Helminth infections such as *Fasciola hepatica*, *Taenia crassiceps*, *Trichinella spiralis*, *Trichinella pseudospiralis*, and *Spirometra mansoni* have been shown to lower inflammatory disorders in EAE animal models [123]. A number of published clinical trials have also embarked upon investigating the utility of helminth immunotherapy for MS treatment. These trials have all involved the administration of *Trichuris suis* ova, a whipworm that establishes persistent infection in pigs but is cleared from the human intestine [124, 125, 126, 127]. In MS patients undergoing helminth therapy, there was a favorable immune modulation profile, with elevated levels of IL‐4, IL‐5, and IL‐10 in the serum, pointing toward a Th2/Treg response. In addition, a reduction in IFN‐γ secretion from stimulated T cells indicated a suppression of autoantigen‐specific Th1 responses [121, 123]. While live parasite usage holds promise for MS patients, it is not considered an ideal approach due to issues surrounding patient compliance and potential side effects. Therefore, a safer alternative strategy involving parasite‐derived molecules from these parasites may present a more reliable option for leveraging their therapeutic benefits.

## 8 Discussion

Mounting evidence points to the impact of modified microbiota composition in MS patients. Variations in gut microbiota are known to influence the host′s immunomodulatory response capabilities. The understanding of how the gut microbiota interacts with immune system and the overall health of the host is a complex matter. Several mechanisms may underlie the influence of gut microbiota on MS, which include the disruption of the intestinal barrier, the association between gut microbiota and the autoimmune response, the impairment of the BBB, and the development of chronic inflammation [128].

Current research highlights the importance of gut barrier integrity in influencing microbial homeostasis and the degree of inflammatory response in MS. A meta‐analysis has shown that MS patients exhibit an increased likelihood of experiencing IBD and gastrointestinal inflammation relative to their healthy counterparts [129]. An increase in the production of toxic metabolites and pro‐inflammatory cytokines could lead to the impairment of the gut epithelial barrier, while the levels of beneficial compounds, including SCFAs and various anti‐inflammatory factors produced by gut microbiota, are diminished. Traditionally, the specific types of SCFAs generated are categorized by bacterial genera. Notable butyrate producers include *Clostridium* clusters IV and XIVa, *Eubacterium*, *Ruminococcus*, and *Faecalibacterium*, whereas acetate and propionate are linked to members of the phylum Bacteroidota. It is important to highlight that propionate can also be synthesized by members of the family Lachnospiraceae and the phylum Verrucomicrobiota. SCFAs function as an essential energy source for the host and its gut microbiota. They are capable of entering the host′s circulation and crossing the BBB, leading to significant effects that extend from their production site. SCFAs contribute to a broad anti‐inflammatory response and help to maintain gut barrier integrity [130]. Evidence also suggests that a decrease in butyrate concentration correlates with signs of elevated gut permeability and a reduction in Tregs present in peripheral blood [44]. Moreover, the lack of isobutyrate and isovalerate is associated with a worsening expanded disability status scale (EDSS) score [131, 132]. The enhancement of gut permeability may also lead to a greater activation of immune cells by gut microbiota in peripheral sites, particularly in the GALT, and promote the transfer of toxic metabolites into the bloodstream [133].

Autoimmune response plays a crucial role in both the onset and progression of MS. In fact, oligoclonal immunoglobulin bands, which result from the intrathecal production of Ig by B cells activated by CNS antigens, are found in the majority of patients at their first presentation, suggesting they may serve as a prognostic indicator for the future development of MS [134]. In comparison to autoantibodies, autoreactive T cells are believed to have a more significant role in the development of MS. It is thought that these T cells become activated outside the CNS, then migrate into the CNS, where they trigger a cascade of immune responses against local autoantigenic tissues. Nevertheless, the precise mechanisms and locations of activation for these autoreactive T cells remain uncertain [135]. The significance of GALT lies in its role in the generation of autoimmune T cells, which is facilitated by the interaction between T cells and the gut microbiota. Autoreactive T cells activated in GALT may be a consequence of molecular mimicry, where microbial elements imitate autoantigens such as MBP, proteolipid protein (PLP), and myelin oligodendrocyte glycoprotein (MOG) [136]. For instance, computer analysis conducted on myelin indicates that it shares molecular similarities with sequences from *Acinetobacter*. Interestingly, MS patients have been found to possess antibodies against *Acinetobacter* [137]. The structural similarities between the MBP and human parvovirus B19 (HPV‐B19) as well as adeno‐associated virus 4 have also been identified. Both MBP and MOG share antigenic similarities with a diverse array of viruses and bacteria, which include *Aspergillus* species, *Lactobacillus*, *Burkholderia*, *Clostridium*, *Schizosaccharomyces*, severe acute respiratory syndrome coronavirus 2 (SARS‐CoV‐2), and some metabolites produced by gut microbiota [138].

It is thought that the disruption of the BBB occurs in the early stages of MS, potentially facilitating the infiltration of leukocytes into the CNS, which may further drive the progression of MS [11]. The integrity of the BBB may be positively influenced by SCFAs, which are important molecules associated with gut microbiota. Butyrate, acetate, and propionate are examples of SCFAs that are primarily produced via the fermentation of dietary fibers by gut microbiota [139]. Components of bacterial cell walls, including lipopolysaccharides (LPS) from Gram‐negative bacteria and lipoteichoic acid (LTA) from Gram‐positive bacteria, may play a crucial role in modifying the expression of tight junctions and the integrity of the BBB by interacting with toll‐like receptors (TLRs) found on the neurovascular system [140]. The formation of chronic inflammation in MS patients involves various immune cell types from both the innate and adaptive immune systems, as well as the cytokines they secrete. Th17 cells, in particular, are identified as key effector cells in the chronic inflammatory process of MS, serving as the main source of inflammatory cytokines like IL‐17, IL‐21, and IL‐22 [141]. The role of gut microbiota is vital in sustaining the balance between pro‐inflammatory and anti‐inflammatory immune responses, as it mediates the development of Tregs and the maturation of Th17 cells [142].

It is likely that various gut microbiota‐based therapeutics will emerge as a key aspect of precision medicine for MS in the near future. To realize this potential, it is essential to gain a comprehensive insight into the unique gut microbial communities of MS patients and to scrutinize how these communities interact with the host′s immune response. Some of these treatments, such as probiotic supplements and customized diet plans, have already been widely used by MS patients because they are easily available. In contrast, therapies like FMT and phage therapy are still in their infancy and require additional preclinical and clinical investigations. An important limitation in gut microbiota research is the depth of sequencing, which has hindered many studies that relied on the 16S marker gene from delivering results at levels below genus or species [143]. Since the functionality of the gut microbiota can differ among strains within a single species, it would be advantageous for future research to identify microorganisms at a more detailed level or to investigate the functionality of the gut microbiome directly. On the other hand, measuring the disease activity and progression of MS presents significant challenges, as the studies incorporated various definitions and time periods, making it hard to draw comparisons between them [144]. Upcoming studies ought to focus on evaluating the long‐term effects of gut microbiota‐based therapeutics, particularly regarding the progression and subtypes of MS. Finally, it is important to take into account certain factors such as age, race or ethnicity, and lifestyle when assessing the composition of gut microbiota, as these factors can significantly influence microbial diversity [145].

## 9 Future Perspectives and Conclusions

Alterations in the human gut microbiome could greatly impact human health and contribute to neurological conditions. The complexity of MS lies in the involvement of intricate networks of various signaling pathways, although a complete understanding of its pathogenesis remains a challenge. Over the past years, a growing body of research indicates a connection between gut dysbiosis and MS. The role of the gut microbiome in the progression of MS is critical, as it directly influences immune system functions, produces vitamins, SCFAs, neurotransmitters, and other metabolites. Considering the essential role of gut microbiota in immune system regulation, interventions aimed at restoring the balance of gut microbiota could represent promising therapeutic strategies for MS. Strategies such as the use of antibiotics, probiotics, vitamins, dietary interventions, FMT, phage therapy, and helminth therapy may serve as potential treatment options. Nevertheless, these therapeutic options should be customized to suit the specific variations in the composition of gut microbiota among the patients. Future studies can also be conducted to assess whether therapies that combine DMTs with microbiota‐based interventions provide greater efficacy than DMTs alone. In addition, the benefits of integrating multi‐omics approaches to predict microbial functions and the molecular mechanisms associated with MS should be highlighted in future research. Undoubtedly, by employing these complementary therapies, we could enter a new chapter in MS treatment, presenting a more affordable, safer, and more readily available option than standard pharmaceuticals. However, future studies should place emphasis on the standardization of these therapies. Finally, it is crucial to prioritize large‐scale studies or trials that thoroughly investigate the practical application of these emerging therapeutic strategies.

## Author Contributions

**Mojtaba Memariani:** conceptualization, project administration, validation, writing – review, and editing. **Hamed Memariani:** data collection, assembly, and writing – original draft.

## Disclosure

All authors have read and approved the final version of the manuscript. Mojtaba Memariani had full access to all of the data in this study and takes complete responsibility for the integrity of the data and the accuracy of the data analysis.

## Ethics Statement

The authors have nothing to report.

## Consent

The authors have nothing to report.

## Conflicts of Interest

The authors declare no conflicts of interest.

## Transparency Statement

The lead author Mojtaba Memariani affirms that this manuscript is an honest, accurate, and transparent account of the study being reported; that no important aspects of the study have been omitted; and that any discrepancies from the study as planned (and, if relevant, registered) have been explained.

## Data Availability

The authors have nothing to report.
